# Applying single‐cell technologies to clinical pathology: progress in nephropathology

**DOI:** 10.1002/path.5417

**Published:** 2020-03-31

**Authors:** Benjamin J Stewart, Menna R Clatworthy

**Affiliations:** ^1^ Department of Medicine University of Cambridge Cambridge UK; ^2^ Cellular Genetics Wellcome Sanger Institute Cambridge UK; ^3^ Cambridge NIHR Biomedical Research Centre Addenbrooke's Hospital Cambridge UK

**Keywords:** single‐cell, scRNAseq, mass cytometry, scATACseq, glomerulonephritis, transplantation, renal cell carcinoma, chronic kidney disease, kidney, nephropathology

## Abstract

Cells represent the basic building blocks of living organisms. Accurate characterisation of cellular phenotype, intercellular signalling networks, and the spatial organisation of cells within organs is crucial to deliver a better understanding of the processes underpinning physiology, and the perturbations that lead to disease. Single‐cell methodologies have increased rapidly in scale and scope in recent years and are set to generate important insights into human disease. Here, we review current practices in nephropathology, which are dominated by relatively simple morphological descriptions of tissue biopsies based on their appearance using light microscopy. Bulk transcriptomics have more recently been used to explore glomerular and tubulointerstitial kidney disease, renal cancer, and the responses to injury and alloimmunity in kidney transplantation, generating novel disease insights and prognostic biomarkers. These studies set the stage for single‐cell transcriptomic approaches that reveal cell‐type–specific gene expression patterns in health and disease. These technologies allow genome‐wide disease susceptibility genes to be interpreted with the knowledge of the specific cell populations within organs that express them, identifying candidate cell types for further study. Single‐cell technologies are also moving beyond assaying individual cellular transcriptomes, to measuring the epigenetic landscape of single cells. Single‐cell antigen‐receptor gene sequencing also enables specific T‐ and B‐cell clones to be tracked in different tissues and disease states. In the coming years these rich ‘multi‐omic’ descriptions of kidney disease will enable histopathological descriptions to be comprehensively integrated with molecular phenotypes, enabling better disease classification and prognostication and the application of personalised treatment strategies. © 2020 The Authors. *The Journal of Pathology* published by John Wiley & Sons Ltd on behalf of Pathological Society of Great Britain and Ireland.

## Introduction

Cells represent the fundamental unit of biology, and tissue homeostasis and function in multicellular organisms requires complex interactions between diverse cell types that ultimately determine organ‐specific phenotype and function. Accurate characterisation of phenotypic heterogeneity, intercellular signalling networks, and the spatial organisation of cells within an organ are crucial to deliver a better understanding of the cellular mechanisms underpinning physiology, and the perturbations that lead to disease. The use of microscopy to examine cellular morphology, together with techniques that allow the detection of immune activation in organs, has formed the cornerstone of tissue‐based disease diagnostics. The application of these imaging techniques, in combination with the measurement of circulating immune cells and biomarkers, has provided a framework for disease classification and staging, and yielded insights into pathogenesis. In this review, using nephropathology as an exemplar, we examine how the rapid expansion of single‐cell technologies is transforming our understanding of disease states, and consider its potential for future translation to clinical practice.

## Current nephropathology practice and limitations

**Figure 1:**
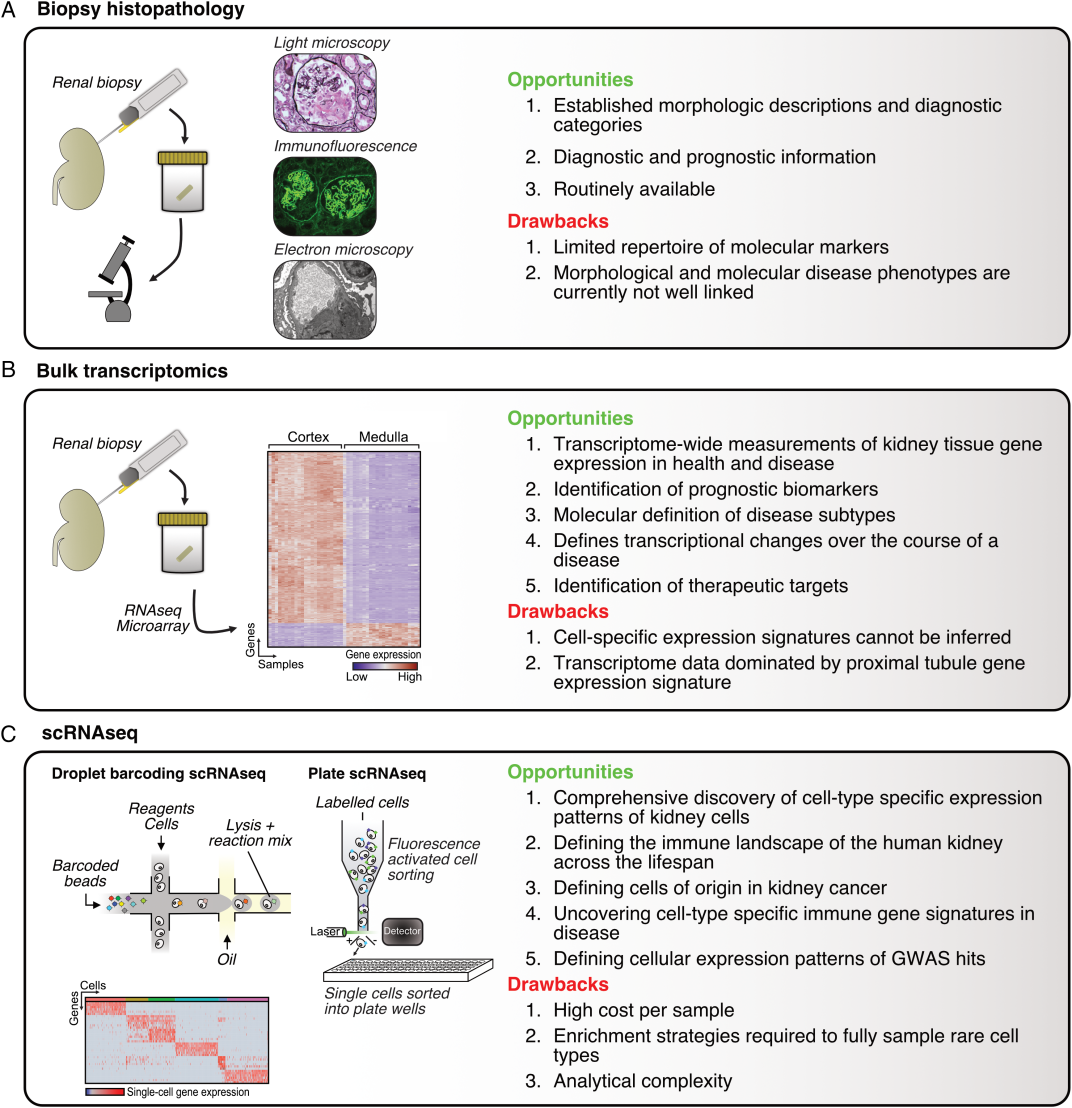
Overview of clinical and experimental methods in nephropathology. (A) Conventional nephropathology relies primarily on morphologic descriptions of renal cortical biopsies. These are examined using a combination of light microscopy (image showing anti‐GBM disease), immunofluorescence (image showing anti‐GBM disease), and electron microscopy (image showing mesangial deposits in immune‐complex mediated glomerulonephritis). (B) Bulk transcriptomics approaches measure aggregate transcript levels in kidney tissue. Image shows genes differentially expressed between samples from cortical and medullary regions from Lindgren *et al* 2017 37. (C) Both droplet barcoding (left) and fluorescence‐activated cell sorting into plate wells (right) can be used to generate scRNAseq data. The heterogeneity of the tissue can be uncovered at single‐cell resolution. Heatmap shows gene expression of single human blood dendritic cells from Villani *et al* 2017 52.

The investigation of renal disease relies on biochemical and immunological analysis of blood and urine, and histopathological examination of biopsy specimens. Non‐invasive diagnostics can yield important information, and in some cases, obviate the need for invasive tissue sampling. However, specific circulating biomarkers of disease are available for only a small subset of pathologies, for example, the presence of anti‐phospholipase A2 receptor (PLA2R) antibodies in membranous nephropathy 1, the presence of anti‐myeloperoxidase (MPO) or anti‐proteinase 3 (PR3) antibodies in anti‐neutrophil cytoplasmic antibody (ANCA)‐associated vasculitis, or circulating anti‐glomerular basement membrane (GBM) antibodies in Goodpasture's disease 1, 2. Thus, renal biopsy remains a crucial investigation in nephrology, providing both diagnostic and prognostic information. Biopsies consist principally of cortical tissue that is subsequently evaluated using a combination of light microscopy, immunofluorescence, and electron microscopy (Figure 1A). Typically, the acquisition of a biopsy is prompted by biochemical analysis of blood demonstrating impaired excretory function of the native kidneys or allograft, often in the context of abnormal urinalysis (haematuria or proteinuria).

In native kidney biopsies, underlying pathologies include autoimmunity, viral or bacterial infection, metabolic disease (such as diabetes mellitus), or genetic disorders. Each are characterised by typical morphological changes within the glomerular, vascular, interstitial, and tubular compartments that allow a diagnostic category to be assigned. For example, a thickened glomerular basement membrane is consistent with a diagnosis of membranous glomerulonephritis (GN). At the ultrastructural level, electron microscopy can identify disease‐associated morphological changes, including podocyte foot‐process effacement and aberrant deposition of immune complexes or fibrils. Immunostaining supplements these data, providing information on the presence and location of specific molecular features. For example, linear glomerular immunoglobulin G (IgG) deposition in anti‐GBM disease, or ‘full house’ immunostaining in lupus nephritis. In transplantation, renal biopsies have a central diagnostic role in the context of allograft dysfunction. The consensus classification system for allograft biopsies is the Banff criteria 3. Biopsy appearances are categorised according to the pattern of renal injury and diagnostic subsets include antibody‐mediated rejection (ABMR), T‐cell–mediated rejection (TCMR), and interstitial fibrosis and tubular atrophy. Immunostaining allows the detection of C4d, indicative of complement fixation by donor‐specific antibodies (DSAs), supporting a diagnosis of ABMR. However, although valuable in classifying appearances into historically defined diagnostic categories, these approaches do not provide insights into the cell‐specific molecular processes that drive disease pathogenesis.

Malignant diseases of the kidney are also classified according to histological appearance. Renal cell carcinomas (RCCs) are the principal neoplasm to affect the kidney parenchyma, and there is considerable heterogeneity within this diagnostic category. RCCs are classified into clear cell RCC (ccRCC), papillary RCC (pRCC), and chromophobe RCC (chRCC), with ccRCC being the most prevalent 4. This classification system is based on histology and characteristic chromosomal alterations 4, 5. In addition, there is heterogeneity within tumour types in terms of oncogene and tumour suppressor gene mutational status. A small proportion of RCCs (2–3%) arise in the context of a hereditary genetic syndrome, for example von Hippel–Lindau (VHL) disease 6. Aside from diagnosis, histological evaluation of cancer biopsies can also provide prognostic information and guide treatment. There is an increasing appreciation that the presence of immune cells within tumours may have prognostic value; for example, infiltration of the tumour with exhausted CD8^+^ T cells and Tregs identifies patients with poor prognosis 7.

## Bulk transcriptomics analysis of kidney tissue: Setting the stage for single cell approaches

Over the last decade, high‐throughput genomics technologies have brought revolutionary new insights into a range of clinical research questions. Transcriptomic technologies including DNA microarrays and RNA‐seq (using next generation sequencing technology), have allowed scientists to assay the full set of RNA transcripts present in a biopsy. Such a measurement represents a bulk average expression of all the cell types within the tissue. Although this information lacks cell‐type specificity, the patterns of gene expression can suggest the presence and even proportions of particular cell types, and the gene expression programmes at play. Furthermore, samples can be clustered in an unbiased manner, on the basis of their global gene expression signatures, revealing disease‐specific transcriptional patterns, and potentially identifying subsets of disease, with implications for targeted therapies or prognosis (Figure 1B).

### Biopsy transcriptomics in benign renal disease

Application of transcriptomics to kidney biopsies has provided valuable insights into a range of renal diseases 8. Much work has focussed on focal segmental glomerulosclerosis (FSGS), a disease characterised clinically by nephrotic syndrome resulting from podocyte injury and the subsequent development of sclerotic glomerular lesions. Microarray analysis of microdissected glomeruli revealed downregulation of markers of differentiated podocytes, and increased transforming growth factor β (TGF‐β) signalling. This study also highlighted the role of leukocyte recruitment, with chemokine genes *CXCL1*, *CXCL2*, *CCL3*, and *CXCL14* found to be upregulated in FSGS samples 9. Indeed circulating leukocytes may be an important aspect of the pathogenesis of FSGS: Work in the mouse models suggests that the circulating factor soluble urokinase‐type plasminogen activator receptor (suPAR)—proposed as a pathogenic permeability factor acting on the podocyte 10, 11 —is produced by bone marrow–derived Gr‐1^lo^ immature myeloid cells. Indeed transfer of these cells induces proteinuric kidney disease in healthy mice 12.

In addition, transcriptomics can yield prognostic information and identify disease biomarkers. Ju *et al* used renal biopsies from four tissue banks to derive non‐invasive biomarkers for chronic kidney disease, including *EGF. EGF* kidney transcripts correlated tightly with estimated glomerular filtration rate (eGFR) and urinary EGF (uEGF). Furthermore, urinary EGF (uEGF) correlated with interstitial fibrosis, tubular atrophy, and the rate of eGFR loss 13. The EGF protein is a tubular‐derived mitogenic protein that modulates responses to injury, promoting repair and regeneration of nephrons 14. These findings have been replicated in a cohort of diabetic patients 15, with uEGF in archived urine samples predictive of chronic kidney disease (CKD) progression in children 16.

More recently, renal biopsy transcriptomics have been applied to study the molecular characteristics of kidney aging. Rowland *et al* studied a 206‐biopsy data set from the TRANScriptome of renaL humAn TissuE Study (TRANSLATE), and validated their findings in publicly available data sets. They identified a gene set associated with kidney aging, including *EGF* and several other genes with predominantly tubular expression, such as the monocarboxylate transporter gene *SLC16A5*, and the Na^+^/K^+^ transporter subunit gene *ATP1B2*. Furthermore, *TSPYL5* expression appeared to be under both genetic and epigenetic control, influenced by a single nucleotide polymorphism (SNP) and CpG methylation 17.

One limitation of bulk transcriptomic analysis of human kidney biopsies is that proximal tubular cells are the numerically dominant cell type in kidney parenchyma; therefore tubular cell gene expression dominates the transcriptome. To overcome this limitation, specific anatomical regions can be microdissected to generate compartment‐specific transcriptional profiles, or gene expression patterns mapped in a biopsy depth‐ and compartment‐specific manner 18. These data sets served as a useful reference for the analysis of subsequent complex scRNAseq data sets 19, 20. More recently investigators have made progress into understanding expression quantitative trait loci (eQTLs) operating in the human kidney in a compartment‐specific manner. Building on efforts to map eQTLs from bulk renal transcriptomics data 21, compartment‐specific eQTL analyses of nephrotic syndrome biopsies identified a range of glomerular‐ and tubulointerstitial‐specific eQTLs. Integrating these data with scRNAseq data established that the expression of genes associated with glomerular or tubulointerstitial eQTLs was enriched in podocytes and proximal tubular cells, respectively 22. Applying a similar approach to chronic kidney disease, Qiu *et al* microdissected 151 human biopsies into glomerular and tubular compartments. Within the tubular compartment they identified an eQTL in the *DAB2* gene specific to the tubular compartment. The associated SNP was also found to be a significant hit in a CKD genome‐wide association study (GWAS). In the scRNAseq data, this gene exhibited selective expression in proximal tubular cells. They concluded that this gene, encoding an adapter for the TGF‐β pathway, is an important novel player in CKD 23.

### Biopsy transcriptomics in transplantation

Molecular diagnostics have been applied extensively to transplantation, with microarray data showing distinct transcriptional patterns in patients with TCMR and ABMR, enabling a more accurate identification of the disease process present in renal transplant biopsies. Reeve *et al* used microarray data from 1208 transplant biopsies and applied the archetypal analysis method to generate probabilistic assessments of biopsy rejection categories. Overall, they were able to identify six biopsy categories, distinguishing biopsies with no rejection, early and late stage ABMR, TCMR, or a small group with mixed TCMR and ABMR. This molecular diagnostic approach showed some discrepancies with histological diagnosis. Indeed, of the biopsies with no histological rejection, 16% had ‘molecular rejection’ according to the transcriptomic readout 24. Of interest, in a separate cohort of transplant biopsies with paired transcriptome profiling and conventional histological assessment, a molecular ABMR score enhanced the power of histological assessment in determining time to graft loss 25.

These studies have also yielded additional insights into pathogenesis, not readily appreciated by light microscopy, for example, natural killer (NK) cell and macrophage genes are enriched in DSA+ humoral rejection 26, 27. Indeed, cellular deconvolution of bulk transcriptomic biopsy data confirmed an association of NK cell signature genes with worse outcomes in ABMR biopsies 27.

Several studies have used allograft biopsy transcriptomics to make inferences about dynamic alloimmune and reparative responses at play within the kidney allograft over post‐transplant time. The large Genomics of Chronic Allograft Rejection (GoCAR) study used biopsy transcriptomics to predict injury due to fibrosis in kidney allografts. Studying 204 biopsies taken 3 months following transplantation from patients with stable renal function, they identified a core set of 13 genes predictive for the development of fibrosis at 1 year. Many of these genes are known to be involved in cell growth and developmental pathways, and are expressed highly in fibroblasts, suggesting that activation of repair processes after an initial insult are associated with kidney fibrosis 28.

Analysis of an additional set of kidney allograft protocol biopsies taken at a variety of timepoints post‐transplant demonstrated an early transcriptional response to ischaemia–reperfusion, followed by bifurcating trajectories toward either healthy recovery or fibrosis and renal dysfunction. Genes upregulated along the fibrotic trajectory included fibroblast‐specific genes such as *COL1A2* and *DCN*, but also chemokine genes such as *CCL19* and *CCL20*, implicating leukocyte recruitment as a feature of progression to chronic graft injury 29. Consistent with this, these authors inferred an accumulation of B cells over post‐transplant time from the correlated expression patterns of B‐cell signature genes. B‐cell gene expression was prominent in samples with chronic allograft fibrosis, and using a murine model of chronic injury after ischaemia‐reperfusion, there was an accumulation of clonally expanded B cells in tertiary lymphoid structures within the kidney. These findings suggest that B‐cell activation and recruitment operates reciprocally with tissue fibrosis responses, culminating in chronic allograft dysfunction 30. Although the transcriptional assessment of transplant biopsies has proved useful in broadly profiling the temporal patterns of gene expression after transplantation, it cannot definitively establish the cell‐type–specific contributions to pathological processes, or determine if gene signatures are of donor or recipient origin.

### Biopsy transcriptomics in kidney cancer

Interrogation of gene expression patterns in renal cell carcinoma has been pursued through The Cancer Genome Atlas (TCGA) 31, 32. This landmark data set revealed transcriptional differences between tumour subtypes that relate to characteristic chromosomal aberrations, and also important insights into cellular metabolic state. In aggressive ccRCCs, there is a switch away from Kreb's cycle gene expression toward glutamine transport and fatty acid synthesis, consistent with the adoption of a ‘Warburg effect’ in cancer cells 31. These data have also provided insights into the immune phenotypes of renal cancers. Overall, ccRCCs show higher expression of immune signature genes than pRCCs or chRCCs, and Th2 skewing of the immune response across all subtypes correlated with poor survival 33. At the level of single genes, expression of *PDCD1* (encoding the T‐cell checkpoint receptor PD‐1) and *CTLA‐4* (encoding CTLA‐4, a T‐cell costimulation inhibitor), also marked tumours with a poor patient survival 34. This was a crucial finding, providing a robust rationale for the use of dual‐checkpoint blockade with nivolumab and ipilimumab in RCCs 35. More recent data also implicate the presence of B‐cell aggregates in tertiary lymphoid structures in the efficacy of checkpoint blockade inhibitors 36.

The cellular origins of different subtypes of RCCs remain an important question that can be informed by comparing the transcriptomes of normal and malignant tissue. Lindgren *et al* identified modules of genes within the normal tissue TCGA data that matched segment‐specific, or compartment‐specific, gene expression patterns in rat and human nephron, respectively. These modules represented coarse ‘cell‐type’ gene expression profiles. Applying these signatures to cancer data, they found a *FOXI1*‐driven signature in chRCCs, suggesting they arise from intercalated cells of the collecting duct, and an *HNF1*‐driven signature in ccRCCs and pRCCs, suggesting that these arise from proximal tubule precursor cells 37.

## Single‐cell technologies

### Flow‐ and mass‐cytometry

Flow cytometry enables cell populations to be studied at single‐cell resolution and has proved revolutionary for cell‐type classification and inferring functional heterogeneity—particularly in immunology and haematology where it is used routinely. This includes the diagnosis of haematological malignancies, primary and secondary immunodeficiencies, and some benign haematological pathologies (such as paroxysmal nocturnal haemoglobinuria, inherited platelet disorders, and in titration of ATG dosing for immunosuppression) 38. However, although flow cytometry has many strengths, such as a wide dynamic measurement range, high throughput, and capacity for specialised measurements (such as intracellular phospho‐signalling, proliferation assays, and calcium flux assays), the number of markers that can be simultaneously measured is limited by spectral overlap of fluorochromes.

In contrast to flow cytometry, which utilises fluorescently labelled antibodies, the more recently developed technology mass cytometry uses antibodies conjugated with heavy metals, which are measured in a mass spectrometer 39. This minimises, but does not eliminate, signal overlap, and allows the simultaneous measurement of up to 40 markers. In theory, this number may extend to around 100 markers, but the limitation in its current use is antibody and isotope availability. Mass cytometry is inherently destructive, and so in contrast to flow cytometry, cannot be used for sorting cells on the basis of surface phenotype. Furthermore, when compared to flow cytometry, mass cytometry has a lower throughput per unit time, and more of the sample is wasted.

Although generating single‐cell data at scale, both flow cytometry and mass cytometry suffer from the bias of antibody availability and marker selection. Although these methods can distinguish populations of cells on the basis of a selected repertoire of markers, these assays do not permit a truly unbiased or comprehensive assay of cellular heterogeneity.

The advent of mass cytometry brought a set of distinct analytical challenges, many of which are present in high‐throughput scRNAseq data. These data are ‘high dimensional’. A large number of features are measured on each cell, and conventional biaxial gating is therefore impractical. Furthermore, the data have the ‘curse of dimensionality’—distances between data‐points become progressively greater with more features, frustrating conventional clustering algorithms. For these reasons, investigators have turned to visualisation methods, which aim to generate a two‐dimensional embedding that faithfully represents the high‐dimensional distances between cells, and the local and global structures of the data 40, 41. In addition, methods have been developed to assign cells to clusters that represent putative populations, modelling the high‐dimensional manifold as a graph structure, and aiming to find well‐connected communities within this graph 42, 43, 44. This approach has also spurred the development of numerous ‘pseudotime’ algorithms, which attempt to order cells captured during a snapshot according to the progress along the trajectory of a biological process (for example, differentiation or activation) 45.

### Single‐cell RNAseq

Over the past decade, scRNAseq has been widely adopted as a powerful tool to understand the heterogeneity and diversity of cells within a sample in an unbiased manner. The approach has scaled from early experiments involving in‐depth characterisation of a handful of cells, to the contemporary generation of atlas‐scale data sets, comprising in excess of hundreds of thousands of cells 46.

The earliest scRNAseq experiments used manual capture of single cells 47, before the field moved to an integrated fluidic circuit approach 48. Currently two broad technical approaches are in widespread use. First, plate‐based scRNAseq in which single cells are partitioned into individual wells of 96‐ or 384‐well plates before reverse transcription and sequencing (Figure 1C). Methods using this approach include the MARseq protocol, which for the first time demonstrated the capacity of scRNAseq to recover the transcriptional identities of cell types within a whole organ, generating profiles of murine splenocytes and their responses to LPS stimulation 49. The SmartSeq2 (SS2) protocol is an alternative plate‐based strategy 50, with the advantage that it generates full‐length transcript data but with a lower throughput. SS2 is therefore suited to uncovering the heterogeneity within a defined population of cells. Full‐length transcript information opens the possibility of uncovering splice variant usage; however, such analyses remain at an early stage of development 51. In a landmark paper demonstrating the utility of this method, Villani *et al* performed SS2 scRNAseq on dendritic cells (DCs) and monocytes sorted from human blood based on known markers. This study revealed previously unappreciated heterogeneity among these cells, including the identification of a novel DC subset expressing *AXL and SIGLEC6*, termed ‘AXL DCs’ 52. In contrast to these focused approaches, which do not explicitly barcode cells, an alternative plate‐based approach—SPLiTSeq—performs multiple rounds of splitting cells into plate wells at random, ligation with barcode oligonucleotides, and pooling to generate unique cellular barcode sequences. The pioneers of this approach were able to produce a comprehensive cell type atlas of the murine brain and spinal cord comprising >100 000 cells 53.

The second major approach is droplet‐encapsulation scRNAseq, in which single cells are incorporated into droplets along with barcoded beads. The reverse transcription reaction occurs within the droplets before lysis, generating barcoded transcripts for pooling to generate highly multiplexed cDNA libraries (Figure 1C). Initially used to characterise heterogeneity among thousands of embryonic stem cells 54, this approach is now capable of delivering massive‐throughput scRNAseq data sets 55. However, the data generated from this approach suffer from relative sparsity, and the formation of technical doublets when two cells are encapsulated in a single droplet. This method has been used to generate large‐scale scRNAseq atlases of human kidneys across development and lifespan 19, 20, 56, 57.

### Single‐nucleus RNAseq

In contrast to single‐cell RNAseq, single‐nucleus RNAseq (snRNAseq) captures only the transcripts present in the nucleus. Nuclei can be prepared from frozen or fresh tissue without the need for tissue dissociation. Although snRNAseq captures fewer transcripts, it avoids tissue dissociation–associated cell stress responses that may generate transcriptional artefacts, and loss of information due to a failure of enzymatic digestion to recover certain cell types 58. snRNAseq is easily incorporated into droplet‐encapsulation pipelines, and has been used effectively on human kidney samples 56.

### Single‐cell assay for transposase‐accessible chromatin sequencing (ATACseq)

The gene expression programmes dictating cellular identity are controlled by regulatory mechanisms operating on the genome such as the chromatin state. Genes may be expressed if their chromatin exists in an open state. scATACseq allows the identification of regions of DNA accessible to the transcription machinery with single‐cell resolution 59. This method has been adapted to perform high throughput droplet‐encapsulation ATACseq on tens of thousands of single nuclei 60, and has been used to chart dynamic shifts in chromatin accessibility during human haematopoiesis, and in basal cell carcinoma of the skin 61. Although this technology remains relatively immature compared to scRNAseq, already scATACseq data on the murine kidney has provided insights into the chromatin regulatory states associated with major kidney cell types 62, 63.

## Defining the immune landscape of the human kidney

The human kidney is an anatomically complex structure. Within the kidney, hundreds of thousands of functional nephron units are arranged over cortico‐medullary‐pelvic depth and are accompanied by specialised vascular beds along distinct nephron segments. Glomerular endothelium forms a tight connection with podocytes of the glomeruli, and ascending and descending endothelium of the vasa recta accompany the loop of Henle in the medulla. Each anatomical region is associated with unique environmental features. Gradients of salinity and hypoxia exist between the cortex and the medulla. Furthermore, the dominant immune challenge in each region is distinct: the cortex is exposed to circulating factors, including immune complexes, whereas the pelvis and medulla are sites of exposure to ascending bacterial infection. We have previously demonstrated that this gradient of salinity orchestrates the recruitment of antibacterial macrophages to the medulla via epithelia‐derived signals to counter this infectious threat 64. Recently, we have profiled the immune compartment of the human kidney in both development and maturity, and uncovered a heterogeneous landscape of leukocytes resident in the kidney. These included a population of M2 (anti‐inflammatory) polarised tissue‐resident macrophages, likely seeded to the kidney early in fetal life 19. These cells expressed markers consistent with macrophage populations identified in normal tissue samples profiled in a mass cytometric atlas of renal cell carcinoma 65. The kidney also harbours monocyte‐derived myeloid populations, T cells, B cells, and heterogeneous populations of NK cells. Probing the non‐immune compartment within these scRNAseq data, we systematically interrogated ligand‐receptor interactions and uncovered epithelia‐derived chemokine signals predicted to attract neutrophils and antibacterial macrophages to the pelvic region. Indeed, in a murine model of pyelonephritis, neutrophils accumulate in the pelvic region of the kidney. Notably, this regionally localised immune defence appears to develop postnatally, as the signals directing this antibacterial defence were not expressed in fetal pelvic epithelial cells 19.

Smaller scale scRNAseq data sets in murine kidney have corroborated this atlas of tissue‐resident immune populations in the human kidney. Using scRNAseq of the mouse kidney, Park *et al* identified a diverse range of immune cells, including macrophages, T and B lymphocytes, and NK cells, alongside major subsets of endothelial and nephron epithelial cell types 66.

Other studies have focussed on kidney development, tracing trajectories of cellular differentiation toward major nephron cell types. Here too, populations of tissue‐resident macrophages seeding the kidney early in development can be discerned 67, 68, 69. Taken together these data are consistent with a model established in murine fate mapping experiments, that resident macrophages in the kidney can be seeded early in life, and coexist in the mature kidney with monocyte‐derived macrophages 70, 71. These early seeded cells likely have ‘accessory’ non‐immunological functions in supporting tissue homeostasis or modulating disease processes 72.

## Exploring cellular complexity in human cancers

Much effort has been dedicated to atlasing tissues, including the kidney, at a single‐cell resolution 73. Characterising the transcriptional networks that define cellular identity provides a framework for understanding the cellular origins of malignant disease, assuming that partially de‐differentiated cells retain a gene expression signature of their cell of origin. Using droplet‐encapsulation scRNAseq of the human kidney in both development, maturity, and in malignancy (Wilms' tumours, ccRCCs, and pRCCs), malignant cells were identified on the basis of somatic copy number variants in the RNAseq data, and corroborated using whole‐genome sequencing. Measuring transcriptional similarity with a classification model trained on data from healthy tissues, the fetal origins of Wilms' tumour cells were confirmed, whereas ccRCCs and pRCCs derived from a specific subtype of mature proximal tubule marked by the expression of *VCAM1* and *SLC17A3* 20.

One key contemporary challenge for single‐cell omics approaches in the study of cancer is matching genetic status of malignant cells with their heterogeneous transcriptional profiles. scRNAseq can infer copy number variation 74, 75, but identifying somatic mutations and reconstructing the evolutionary relationships of malignant cells from these data is currently not possible. Future work is therefore likely to integrate multiple omics methods to discern the relationship between tumour evolution and transcriptional state and will further leverage the ability of unbiased scRNAseq to provide information on infiltrating and resident non‐malignant cells in the tumour microenvironment.

## scRNAseq applied to kidney biopsy samples

scRNAseq has also been used to investigate tissues affected by disease. Kidney and skin biopsies donated by patients with lupus in the AMP RA/SLE Consortium identified an interferon‐inducible gene signature in renal tubular cells, which was also present in keratinocytes. These data have suggested that skin biopsy transcriptomics could be utilised as a biomarker for disease severity in lupus nephritis 76. More recently, the approach was scaled up to include paired skin and renal biopsies from 21 individuals yielding 4019 cells. Again these data showed an interferon‐inducible gene signature in both tubular cells and keratinocytes in lupus nephritis patients that correlated with poor responses to treatment 77.

A further study of kidney biopsies from patients with lupus nephritis revealed 21 subsets of leukocytes, with all major subsets displaying an interferon‐induced signature. Within the lymphoid compartment, effector memory T cells, Treg cells, and Tfh‐like cells, plasma cells, and B cells were observed. A subset of B cells expressed an ‘age‐associated B cell’ signature, previously implicated in autoimmunity 77, 78. There was also good concordance between the transcriptomes of leukocytes recovered from urine and those in the kidney biopsy, suggesting that a cellular urinary biomarker of renal autoimmunity may be feasible 79.

In a larger scale study, Wilson *et al* performed snRNAseq on cryopreserved kidney samples from patients with diabetic kidney disease (DKD) and controls, generating 23 980 single nuclear transcriptomes. These data included most major nephron cell types in addition to endothelial cells, mesangial cells, and leukocytes (including monocytes, plasma cells, T cells, and B cells). Here the authors uncovered unexpected insights into renal pathophysiology in DKD, showing that cells from distal nephron segments upregulate genes for potassium secretion such as *WNK1* and the mineralocorticoid receptor. In the proximal nephron, they noted increased angiogenic signalling in podocytes and proximal tubular cells. DKD typically results in the development of glomerular lesions and is accompanied by proteinuria. Within this study, the authors uncovered alterations in ligand‐receptor interactions between podocytes, mesangial cells, and endothelial cells, which may represent the earliest pathophysiological changes of DKD. These included *CCN1* expression by mesangial cells; this ligand is capable of modulating podocyte and endothelial cell integrins, and is known to direct tissue repair and fibrosis 80. Although this study identified leukocytes within the DKD biopsies, in contrast to scRNAseq data from a streptozotocin‐induced eNOS^‐/‐^ murine model of DKD, they did not recover macrophages 81, 82. This may reflect a species‐specific difference or indicate that macrophage accumulation occurs in response to the substantial injury induced in the murine model, but may not be present in early, mild DKD. This underpins the importance of pairing conventional histological and biochemical analyses with sequencing approaches to better understand the biological significance of transcriptional signatures.

In transplantation, one study has reported single‐cell transcriptomics data from kidney allograft biopsy material, generating data on 8746 cells from a recipient with a diagnosis of mixed rejection. Within the endothelial cell compartment, activated endothelial cells displayed a gene‐expression signature consistent with Fc receptor pathway activation, and the data provided a cell‐type specific transcriptional characterisation of DSA‐induced vasculopathy in ABMR. No macrophages were recovered in this experiment, but two monocyte populations corresponding to classical and non‐classical monocytes were evident 83. The latter had a high expression of CD16 (*FCGR3A)*, conferring the capacity to respond to IgG alloantibodies. This study also illustrates how scRNAseq data may be used to refine conclusions made from bulk transcriptomic experiments; a number of genes previously included in an ‘endothelial cell activation’ signature indicative of ABMR, were, in fact, found to be expressed in podocytes, fibroblasts, and a variety of kidney immune cells, providing important insights into the pathogenic mechanisms underpinning this ABMR‐associated signature.

This study did not delineate donor and recipient cells in the inflammatory infiltrate in the allograft but the differentiation of genotypically distinct cells has been achieved using droplet encapsulation scRNAseq data in a bone marrow transplant recipient using both autosomal 55 and mitochondrial variant status 84. Byrne *et al* used sex‐restricted gene expression patterns in scRNAseq data from sex‐mismatched lung transplant recipients to show that alveolar macrophages in the transplant context are predominantly recipient derived 85. Donor‐ or recipient‐derived cells can also be sorted based on HLA genotype using HLA‐specific antibodies, before profiling using single‐cell genomics 86. In the context of the maternal‐fetal interface, Vento‐Tormo *et al* used reference whole genome sequence data to differentiate maternal and fetal cells in the human decidua 87. Cutting‐edge methods are in development allowing dissection of genotypic chimerism on the basis of variants called directly from scRNAseq data 87, 88, 89. These will be powerful tools to distinguish donor and recipient cells in transplant tissue.

## Matching single‐cell genomics and GWAS data

**Figure 2:**
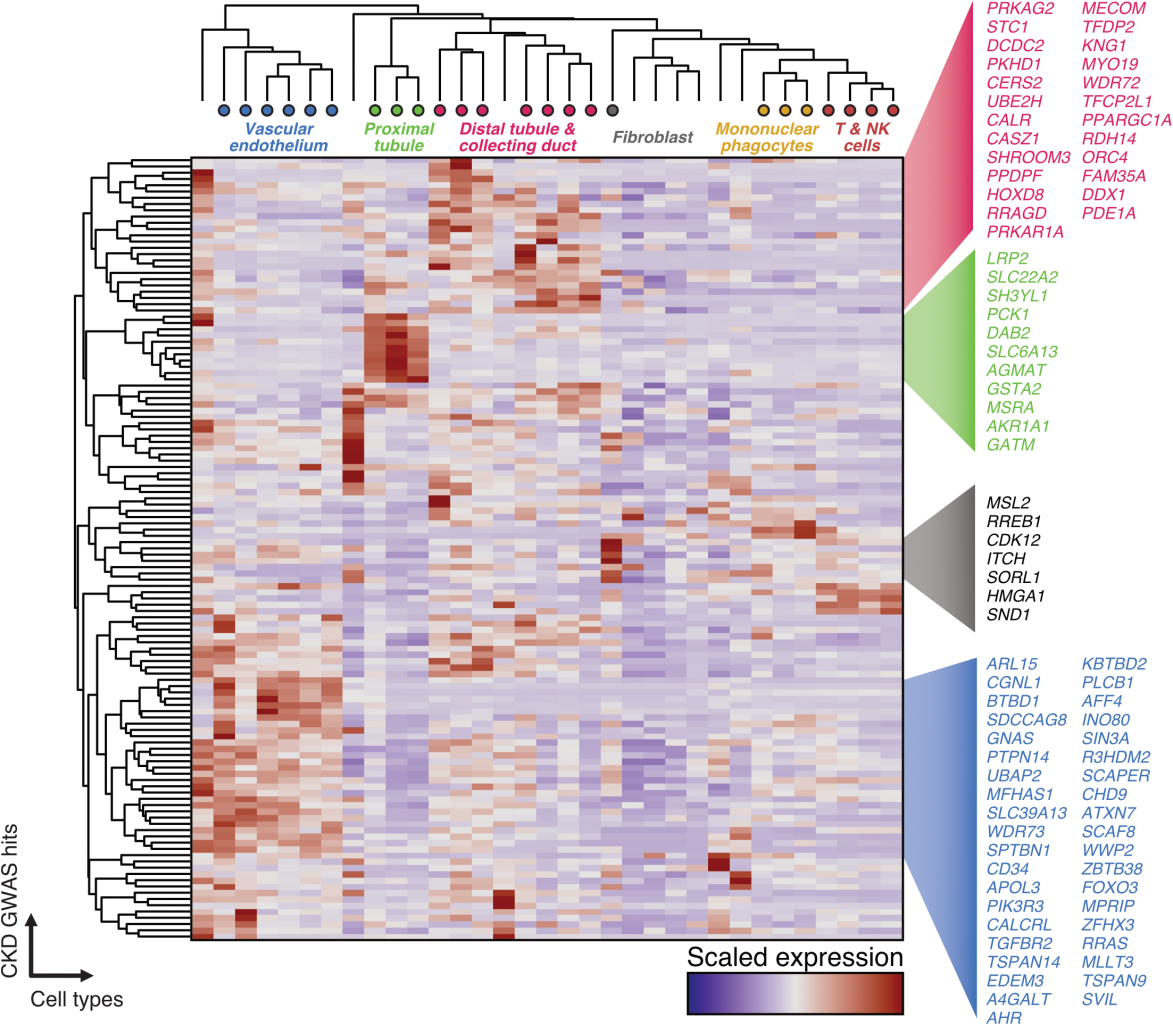
Cellular expression patterns of GWAS hits associated with renal function and chronic kidney disease in human kidney scRNAseq data. Heatmap showing scaled expression values of GWAS hits for renal function and CKD. Cell types and compartments are highlighted, along with sets of genes with cell‐type‐ and compartment‐specific expression patterns.

GWAS identify loci and genes that are associated with disease susceptibility, but give no indication as to in which cell type the gene is expressed to mediate disease pathogenesis. scRNAseq data sets allow the cell expression patterns of genes implicated in GWAS, or indeed monogenic disease‐associated genes, to be precisely mapped. In the kidney, murine scRNAseq data localise the expression of the majority of nephrotic syndrome associated genes to the podocyte, whereas genes associated with hypertension, renal stone formation, and hypertension localised to nephron tubular cell types 66. Combining lupus GWAS hits with their atlas of cell types and states in lupus nephritis biopsies, Arazi *et al* found that the majority of GWAS‐identified genes are expressed in the B cells infiltrating the kidney in disease 79. To date, comprehensive mapping of kidney disease–associated genes to human kidney scRNAseq data sets has not been performed, but utilising data from Stewart *et al*, in combination with results from a large scale GWAS of CKD and renal function traits 90, we mapped variants associated with renal function to kidney cell types (Figure 2). This analysis identified gene sets with specific patterns of cell‐type enrichment—notably proximal tubule—and fibroblast‐specific genes are evident, and genes with compartment specificity including a gene set expressed in vascular endothelium (Figure 2). This illustrates the value of scRNAseq to inform future studies, guiding cell‐type enrichment strategies in disease biopsies for targeted investigation.

## Future directions

As the scale and throughput of single‐cell methodologies increases, these technologies will be applied routinely to clinical samples. Coupling this information with traditional methods of interrogating biopsies will reveal richer descriptions of pathophysiology, and enable data‐driven diagnosis, prognostication, and selection of rational therapeutic strategies.

These technologies are also poised to move beyond transcriptional profiling to enable simultaneous multi‐omic profiling of single cells. Currently single‐cell nucleosome, methylation, and transcription sequencing (scNMTseq) can profile chromatin accessibility, methylation, and transcriptome simultaneously in single cells 91, and this method has already uncovered the global molecular regulation underpinning germ layer formation during murine gastrulation 92. Similarly genome and transcriptome sequencing (G + Tseq) is able to jointly profile single‐cell genomes and transcriptomes 93. In the context of cancer biology, such methods will delineate the clonal architecture of cancers and profile intratumoral heterogeneity.

Current high throughput single‐cell methodologies generate transcriptional information from cells in a suspension produced by disaggregating tissue. These experiments do not preserve information on the spatial relationships between cells, which is critical for normal tissue function. One solution to this problem is reconstruction of spatial arrangements using well‐characterised reference data. This has been effectively achieved in the mammalian liver, where gradients of gene expression in hepatocytes and endothelial cells accompany environmental gradients across liver lobules 94, 95. In organs with more complex parenchymal structures such as nephrons in the kidney, *in situ* spatial transcriptomics offer an opportunity to spatially define patterns of gene expression. These methods can be divided into single‐cell approaches, which measure a smaller repertoire of markers using fluorescent RNA probes 96, or methods that provide a whole transcriptome read out but lack single‐cell resolution 97. Both are likely to play a role in translating insights gained from scRNAseq to a spatial context. Indeed, these methods will be integral to bridging the gap between molecular characterisation of disease using transcriptomics, and classical morphologic approaches to nephropathology, enabling better disease classification and prognostication and the application of personalised treatment strategies.

## Author contributions statement

BJS conducted the reanalysis of human kidney scRNAseq data. BJS and MRC wrote the manuscript.
